# Decoding and spatial mapping of acoustic noise in the neonatal intensive care unit

**DOI:** 10.1038/s41598-025-17707-6

**Published:** 2025-09-01

**Authors:** Hanan Mukhaiber, Kinana Ali, Ebrahim Ismaiel

**Affiliations:** 1 Department of Biomedical Engineering, Faculty of Mechanical and Electrical Engineering, Damascus University, Damascus 86, Syria; 2 Faculty of Mechanical and Electrical Engineering, Tishreen University, Latakia, Syria; 3 Department of Medicine and Surgery, University of Parma, Parma, 43125 Italy

**Keywords:** Neonatal ICU, Noise, Acoustic map, Vocal intensity, Spatial mapping, Paediatrics, Biomedical engineering

## Abstract

The neonatal intensive care unit (NICU) is a critical care setting where premature infants face continuous exposure to elevated noise levels, often exceeding international safety guidelines. While the risks of excessive acoustic exposure are well established, strategies for real-time noise monitoring and mitigation in operational NICUs remain underexplored. In this study, we propose an exploratory framework that integrates spatially distributed sound sensors, acoustic heatmap visualization, and machine learning-based classification to analyze and categorize noise events in a high-density NICU setting. The analysis identified persistent high-noise zones near incubators and entryways, with staff movement and alarm-related activities causing significant sound level spikes—particularly during the noon shift. Additionally, a random forest classifier achieved 85.5% accuracy in distinguishing clinical activity patterns based on environmental acoustic data. While not intended for urgent alerting, this framework demonstrates the potential of using ambient sound profiles for non-critical event recognition and environmental monitoring in the NICU.

## Introduction

The neonatal intensive care unit (NICU) represents a pivotal environment for the survival of premature and critically ill infants^1^, yet it often exposes these vulnerable patients to harmful levels of environmental noise^2–5^. Preterm infants, whose auditory and neurological systems are still developing, are especially susceptible to acoustic overstimulation, which can lead to disruptions in sleep, stress regulation, cardiorespiratory instability, and long-term cognitive and language impairments^6–8^. Numerous studies have reported that routine NICU sound levels regularly exceed safe thresholds, often ranging from 57 to 97 dBA^4,9^, far above international guidelines that recommend maintaining noise within 20–40 dBA by day and ideally closer to 10 dBA during rest periods^10–12^. The U.S. Environmental Protection Agency (EPA) and the American Academy of Pediatrics (AAP) further specify maximum allowable levels of 45 dBA (daytime) and 35 dBA (nighttime)^5,13–15^.

Despite these recommendations, empirical research across different settings has revealed consistent non-compliance^16–23^. Investigations by Fortes-Garrido et al.^17^ and Zamberlan-Amorim et al.^18^ found significant and sustained noise violations in both Spanish and Brazilian NICUs, respectively. Crofts et al.^19^ further showed that even with staff education and intervention based on Plan-Do-Study-Act (PDSA) methodology, average NICU sound levels often remain above safety limits. The persistence of excessive noise is linked to a variety of sources—alarms, conversations, and staff movement—which are notoriously difficult to control and predict^22,23^.

Architectural and spatial design factors significantly shape the acoustic environment of NICUs. Rodríguez-Montaño et al.^20^ and Hernández-Molina et al.^22^ emphasized how physical layout, material selection, and reverberation characteristics influence sound propagation patterns. Comparisons between open-bay and single-family room (SFR) configurations have shown that while SFRs can reduce overall noise levels, they also introduce challenges such as caregiver isolation and inconsistent acoustic control^21,23^. In resource-limited settings, Nathan et al.^24^ highlighted that the absence of sound-dampening infrastructure leads to heightened and sustained reverberant noise exposure.

Beyond simple decibel measurements, recent studies have drawn attention to the developmental and physiological consequences of NICU noise exposure. Bertsch et al.^8^ documented how incubators distort human speech and music, potentially disrupting early auditory learning, while Lasky and Williams^14^ found associations between elevated sound levels and physiological stress in extremely low birth weight infants.

Despite this growing body of evidence, there remains a critical gap between observational research and actionable strategies for sound control, particularly in under-resourced healthcare environments where NICU layouts do not adhere to international standards. This translational shortfall has direct implications for infant health outcomes, especially in contexts where environmental modifications are constrained by physical, infrastructural, or economic limitations.

In this study, we propose a real-world, data-driven framework for acoustic monitoring in a public hospital NICU characterized by high cot density and minimal acoustic regulation. Leveraging a spatial sensor network and temporally annotated clinical activity logs, we apply advanced statistical analysis and machine learning techniques to decode the acoustic signatures of everyday care routines. Our objectives are threefold:

1. to quantify and visualize spatial and temporal distributions of sound pressure levels in the NICU,
2. to characterize clinical events and unit routines most associated with elevated noise exposure, and
3. to suggest and evaluate machine learning models capable of classifying high-risk acoustic scenarios, with the potential to inform anticipatory actions—such as alerting staff to escalating noise levels, guiding the clustering of non-urgent care tasks, or supporting workflow adjustments aimed at reducing repeated disruptions near sensitive infants.

## Methods

### Study setting

This study was conducted in the NICU of the university hospital affiliated with Damascus University, Syria. Ethical approval for the study was granted by Damascus University (Resolution No. 1352, dated 14 April 2015). All methods were carried out in accordance with relevant institutional guidelines and regulations. For this research involving human participants who are minors, informed consent for participation was obtained from a parent and/or legal guardian of each neonate enrolled in the study.

The NICU is located on the second floor of the hospital, adjacent to the delivery and cesarean departments. It has a dedicated main entrance equipped with a bell, which contributed to acoustic events during the study. The unit comprises multiple functional spaces:

1. A general newborn care room (6 × 4 m) with 12 cots,
2. Two critical care rooms, each with four incubators (6 × 4 m and 6 × 5.8 m, respectively),
3. A centralized nurses’ station,
4. A doctors’ office, service area, and two equipment storage rooms.

Our measurements were conducted in one of the critical care rooms. This incubator room includes:

1. Three double-glazed windows to reduce external environmental noise,
2. External wall insulation using aluminum panels (non-acoustic grade),
3. Industrial granite flooring (hard, non-porous),
4. Standard wooden interior doors, and.
5. An internal layout with incubators spaced approximately 50 cm apart, reflecting a high-density configuration.

Each room is equipped with four wall-mounted oxygen and medical air outlets, maintaining constant air pressure at 5 bar. The proximity of cots, the reflective surface materials, and the lack of internal acoustic treatment collectively contribute to a reverberant sound environment, representative of many under-resourced NICUs. Notably, the inclusion of a bathroom within the room, while uncommon in high-income settings, reflects local infrastructure realities and introduced distinct noise events (e.g., door usage, flushing, and water flow).

This setting, while non-compliant with international NICU design standards, provides critical insights into the acoustic challenges faced in constrained clinical environments. It serves as a representative model for data-driven noise risk monitoring in similar global healthcare contexts.

### Equipment and measurement setup

Sound pressure levels were recorded using four identical HTC SL-1352 digital sound level meters, which comply with Class 3 specifications according to IEC 61672-1. These instruments apply A-weighting filters to simulate human auditory sensitivity across frequencies, making them suitable for evaluating environmental noise in clinical settings^25^.

Each sound level meter was mounted at a height of 1.3 m from the floor, following recommendations outlined in the Australian Standard AS 1259 for room acoustics measurement^26^. To enhance directional sensitivity and minimize angular reflection errors, meters were tilted at approximately 70 degrees relative to predominant noise sources, as supported by acoustic measurement protocols^27^.

To capture the spatial variability of noise within the incubator room, the devices were strategically positioned at the four corners of the space.

Sound level readings were recorded in A-weighted decibels (dBA) and stored using the meters’ internal logging function with sampling rate of 1 Hz. Data were downloaded via USB interface, processed using the manufacturer’s analysis software, and exported into Microsoft Excel 2013 for statistical preprocessing. The resulting dataset was used to generate spatial noise distribution maps and served as the primary input for machine learning-based classification of noise events, as detailed in subsequent sections.

### Measurement protocol

Noise measurements were carried out over a continuous 20-day period, encompassing 45 distinct measurement cases designed to capture the full range of acoustic variability under routine NICU conditions. These cases were categorized based on three key operational variables:

1. **Time of day**: morning (07:00–12:00), noon (12:00–17:00), and evening (17:00–22:00),
2. **Occupancy level**: ranging from zero to four neonates in the monitored room,
3. **Activity type**: including quietness periods (minimal human activity), active staff movement, and episodic events such as door openings and medical interventions.

Case definitions were informed by a prior observational audit of NICU operations, allowing for representative selection of measurement scenarios aligned with typical daily rhythms and workflows. The duration of each case ranged from 5 to 25 min, and each was annotated in real time with detailed contextual notes regarding clinical or environmental events.

Importantly, all data were collected passively during standard care activities without interference or alteration to clinical routines. Staff were informed of the study’s general purpose at the outset but were not given specific case timing, minimizing behavioral bias and mitigating the Hawthorne effect. This approach ensured ecological validity, with the goal of producing sound exposure data that accurately reflect the neonatal auditory environment in a real-world high-density NICU.

### Acoustic parameters

The equivalent continuous sound level ($\:{L}_{eq}$) was calculated, providing a single representative value for the cumulative noise exposure over time. The $\:{L}_{eq}$ is determined by the formula:

1 $$\:{L}_{eq}=10{log}_{10}\left(\frac{1}{T}\sum_{i=1}^{n}{10}^{\frac{Li}{10}}\right)$$

where $\:{L}_{i}$ is the measured sound level at a specific moment $\:{t}_{i}$​, and T is the total measurement duration.

The sound level meters were programmed to automatically record $\:{L}_{eq}$ values, but where necessary, manual calculations were performed using the collected time-series sound intensity data. Manual calculations of $\:{L}_{eq}$​ were only required in a small number of sessions (< 5%) where raw time-series dBA values were recorded directly due to temporary logger malfunction or reconfiguration of the sound level meter. In these instances, the automatic computation of $\:{L}_{eq}$​ was unavailable, and we applied Eq. (1) to the recorded 1 Hz dBA samples to reconstruct the equivalent continuous sound level over the specified interval. This ensured consistency across sessions and preserved data integrity in cases where automated summaries were not captured.

The internal summation represents the arithmetic mean of the instantaneous sound intensities (expressed in linear units), which are then logarithmically compressed back into the decibel scale. Since $\:Li$is unitless in logarithmic form, and (1/T) carries units of 1/s, the result of the expression remains dimensionally consistent for $\:Li$ in dBA. In our system, sound level meters were programmed to directly output $\:Li$ values at fixed intervals. However, when manual calculation was required, the time-series dBA data were processed using Eq. 1 to ensure accurate temporal averaging of acoustic exposure.

### Data analysis

#### Spatial acoustic mapping

To assess spatial variability in sound pressure levels within the NICU incubator room, a custom acoustic mapping algorithm was developed using MATLAB. Input data consisted of synchronized dBA measurements collected from four fixed sound level meters placed at each corner of the room. These readings were interpolated across a two-dimensional spatial matrix using bilinear and bicubic interpolation techniques to estimate sound intensity values at unsampled locations. Supplementary virtual grid points were generated to improve mapping resolution. The resulting acoustic field maps were visualized as heatmaps, enabling high-resolution spatial inspection of noise distribution patterns under various clinical and temporal scenarios.

#### Statistical analysis and classification

All statistical and machine learning analyses were performed in Python using the Google Colab environment. The raw dataset was preprocessed to include:

- **Raw dBA readings** from each sensor (A–D),
- **Deviation from the mean (d)** and **squared deviation (d²)** to capture localized noise fluctuations,
- **Time-of-day** and **event-type labels** based on manual annotations,
- **Aggregated metrics** (mean, max, range) across sensors for feature compactness.

To evaluate the influence of NICU conditions on acoustic levels, a one-way ANOVA was conducted, followed by Tukey’s HSD post hoc analysis to detect statistically significant pairwise differences between event categories (e.g., quietness, staff entry, door activity). These analyses quantified how specific activities contributed to changes in the acoustic profile of the room.

#### Machine learning classification

Acoustic data were collected in the NICU over 45 measurement sessions spanning a 20-day period, capturing a range of routine clinical conditions. The recordings were segmented into non-overlapping 5-second windows, corresponding to five samples per segment given the 1 Hz acquisition rate, and averaged to reduce short-term variability and enhance feature stability for subsequent analysis. The raw recordings were pre-segmented, averaged and structured into a dataset containing 8,639 instances (rows), each representing a labeled acoustic event period, with 15 columns including primary sensor readings, their first and second derivatives, and event labels.

Following data cleaning and label consolidation, six clinically relevant acoustic event categories were retained for classification: Quietness, Staff Movement Morning, Staff Movement Noon, Staff Movement Evening, Door Activity, and Alarm. The distribution of labeled samples across these categories is presented in Table 1. For classification, four primary acoustic sensor channels (A, B_sensor, C_sensor, and D) were used as features. The dataset was randomly split into training and testing sets using an 80/20 stratified split to preserve class distribution, resulting in 6,911 samples in the training set and 1,728 samples in the testing set, each with 4 feature dimensions.

**Table 1:** Distribution of labeled acoustic events and dataset dimensions.

Event category	Number of samples
Quietness	3058
Staff Movement Morning	1691
Staff Movement Noon	1691
Staff Movement Evening	1691
Door Activity	280
Alarm	228
Total	8,639
Dataset split	Shape (Samples × Features)
$\:{X}_{train}$	6911 × 4
$\:{X}_{test}$	1728 × 4

To evaluate the potential for automated recognition of clinical acoustic events in the NICU, supervised classification models were trained using the scikit-learn and XGBoost libraries in Python. The target variable consisted of labeled clinical events derived from synchronized annotations, including “Quietness,” “Staff Movement,” “Door Activity,” and “Parent Entry.” Three classifiers were implemented^28^

1. **Random Forest**: Number of trees set to 200, with no maximum depth constraint, a minimum of 2 samples required to split an internal node, and a minimum of 1 sample required at leaf nodes.
2. **XGBoost**: Number of boosting rounds set to 150, maximum tree depth set to 5, learning rate set to 0.1, subsample ratio set to 0.8, column subsample ratio by tree set to 0.8, and evaluation metric specified as multi-class logarithmic loss.
3. **Support Vector Machine (SVM)**: Radial basis function (RBF) kernel used, with a regularization parameter (C) of 1.0, and gamma parameter set to ‘scale’ (automatic adjustment based on the number of features).

While the current implementation was conducted offline, RF and XGBoost were chosen for their ability to handle high-dimensional, non-linear data while providing feature importance for interpretability, and SVM was included as a complementary non-ensemble method effective for non-linear decision boundaries.

Model performance was evaluated using four standard classification metrics: accuracy, precision, recall, and F1-score, and validated through 5-fold cross-validation to ensure robustness and reduce the risk of overfitting. Accuracy quantifies the proportion of correct outcomes over all classifications and is defined as:

2 $$\:Accuracy=\frac{TP+TN}{TP+TN+FP+FN}$$

where $\:TP$ is the number of true positives, $\:TN$ is the number of true negatives, $\:FP$ is the number of false positives, and $\:FN$ is the number of false negatives. Precision, which measures the proportion of correctly identified positive samples among all classified positives, is calculated as:

3 $$\:Precision=\frac{TP}{TP+FP}$$

Recall, also known as sensitivity, represents the proportion of true positives correctly identified among all actual positives:

4 $$\:Recall=\frac{TP}{TP+FN}$$

Finally, the F1-score, which balances precision and recall, is defined as the harmonic mean of the two:

5 $$\:F1-score=\frac{2\times\:Precision\times\:Recall}{\text{P}\text{r}\text{e}\text{c}\text{i}\text{s}\text{i}\text{o}\text{n}+\text{R}\text{e}\text{c}\text{a}\text{l}\text{l}}$$

## Results

**Fig. 1:**
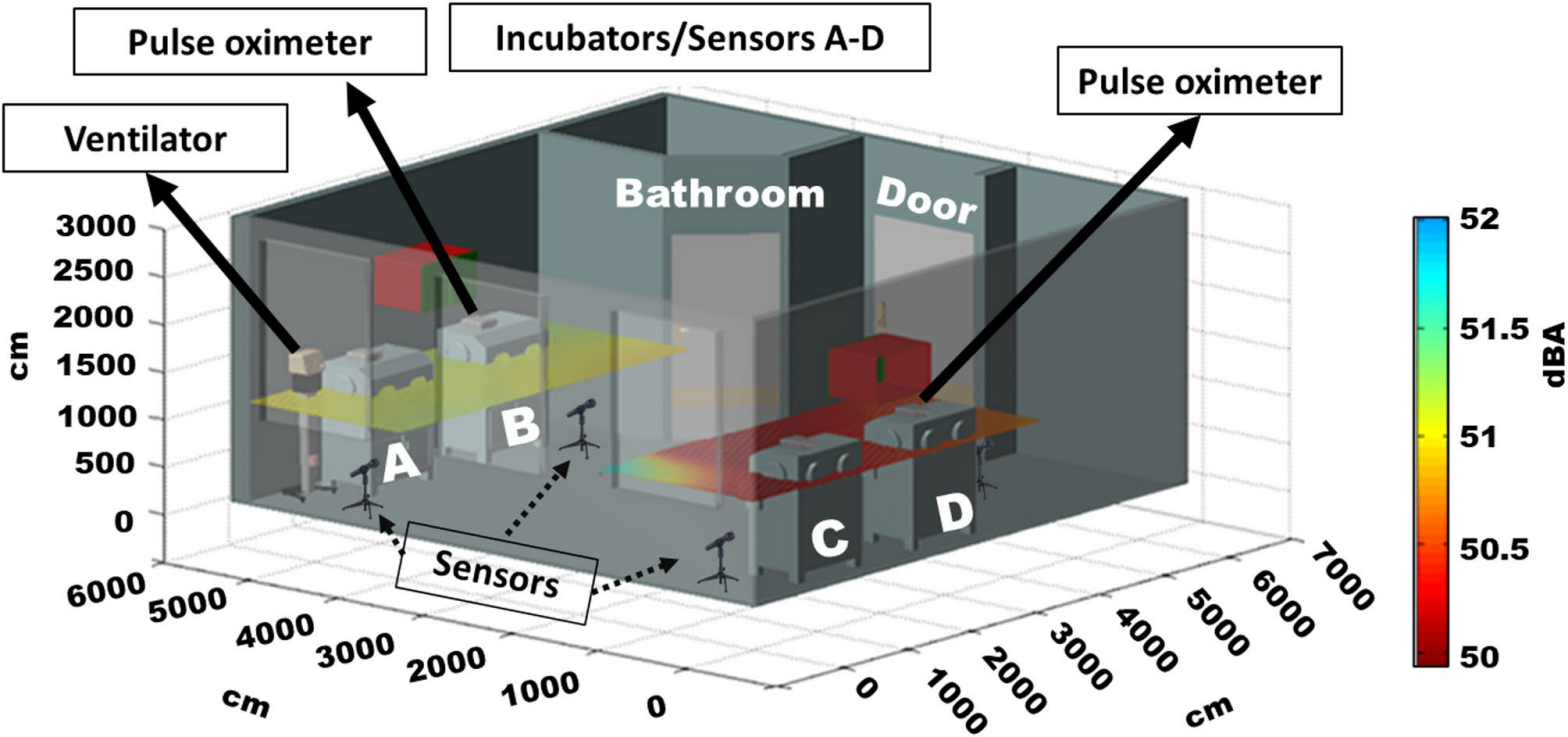
3D schematic of the NICU showing spatial heatmap of average acoustic intensity (dBA) captured by sensors A–D that represent the incubators as well.

To investigate spatial variation in acoustic exposure within the NICU, Fig. 1 presents a three-dimensional schematic of the incubator room overlaid with interpolated heatmaps derived from dBA readings collected by sensors A–D. The spatial heatmap in Fig. 1 reveals that sensors A and B, positioned near the ventilator and doors side, recorded persistently higher acoustic intensities, indicating areas of elevated noise exposure within the NICU environment. This spatial variability aligns with structural factors, such as proximity to entrances and active equipment, highlighting how environmental layout influences localized sound exposure.

**Fig. 2:**
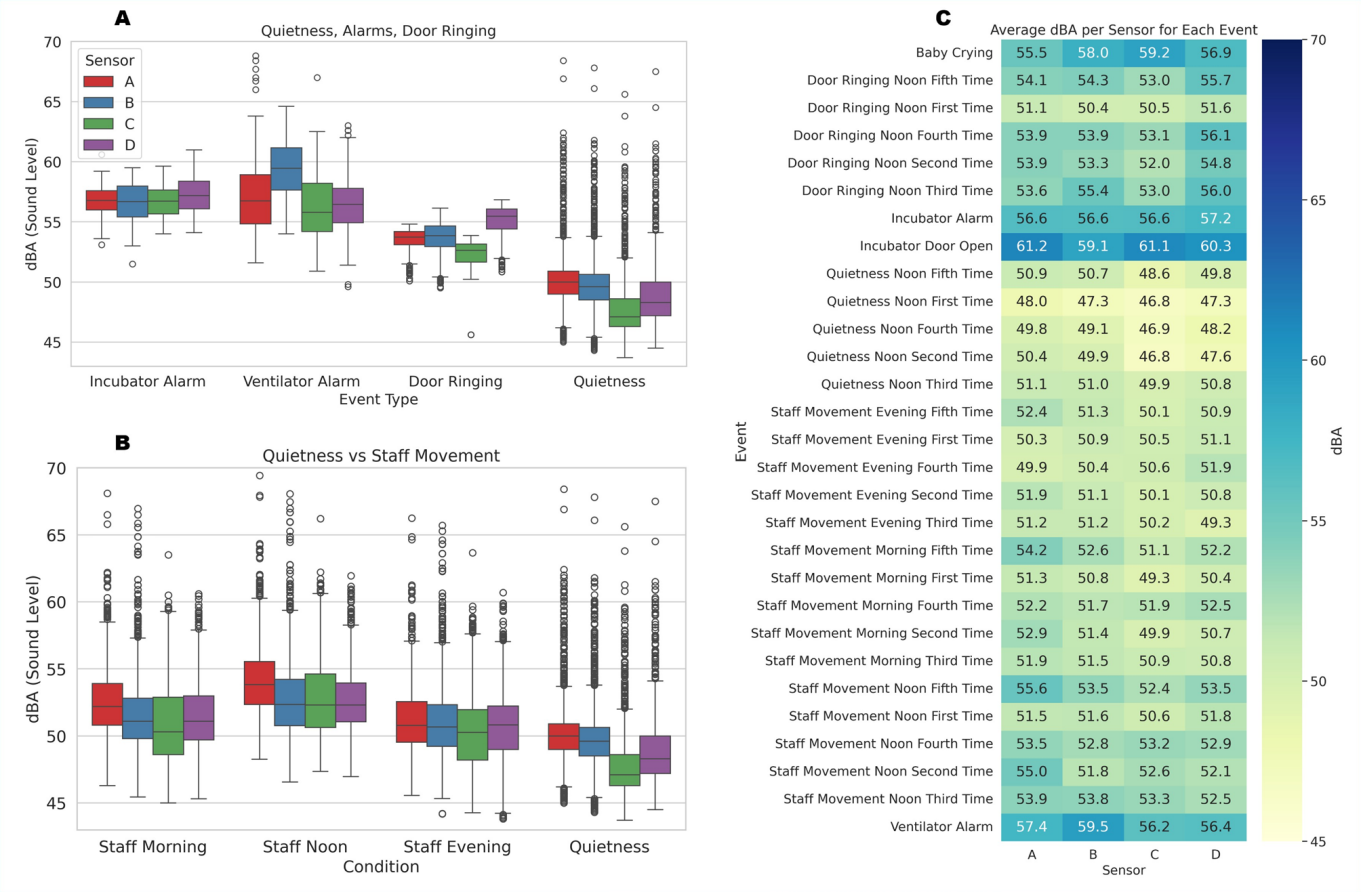
Quantification of NICU acoustic variation across conditions and events. (**A**) Boxplots of dBA levels from sensors A–D during incubator alarms, ventilator alarms, door ringing, and quietness. (**B**) Sound level comparison during staff movement across morning, noon, and evening periods versus quietness. (**C**) Heatmap of average dBA values recorded by each sensor for all monitored events.

Figure 2A illustrates the distribution of dBA levels recorded during incubator alarms, ventilator alarms, door ringing events, and quietness periods. Among these, ventilator alarms generated the highest median sound levels, with values ranging from 56 to 59 dBA across the four sensors. Incubator alarms followed closely, with median levels between 55 and 58 dBA. Door ringing events produced intermediate values, while quietness periods consistently demonstrated the lowest acoustic levels, typically ranging from 49 to 51 dBA.

Figure 2B compares acoustic exposure during staff movement across morning, noon, and evening shifts with quietness conditions. Noon periods were associated with the highest median sound levels, typically between 52 and 55 dBA. Morning and evening staff movements generated slightly lower values but remained elevated relative to quietness. Across all shifts, staff movement resulted in consistently higher sound exposure than the quietness, reaffirming the contribution of human activity to NICU noise elevation. Figure 2C demonstrates that sensor A frequently recorded the highest average dBA values across most monitored events, particularly during alarm conditions and certain staff movement periods, followed by other sensors. This reflects the influence of local event occurrences and the proximity of sensor A to noise sources, such as the pulse oximeter, while sensors B and C generally recorded lower levels across most conditions.

**Fig. 3:**
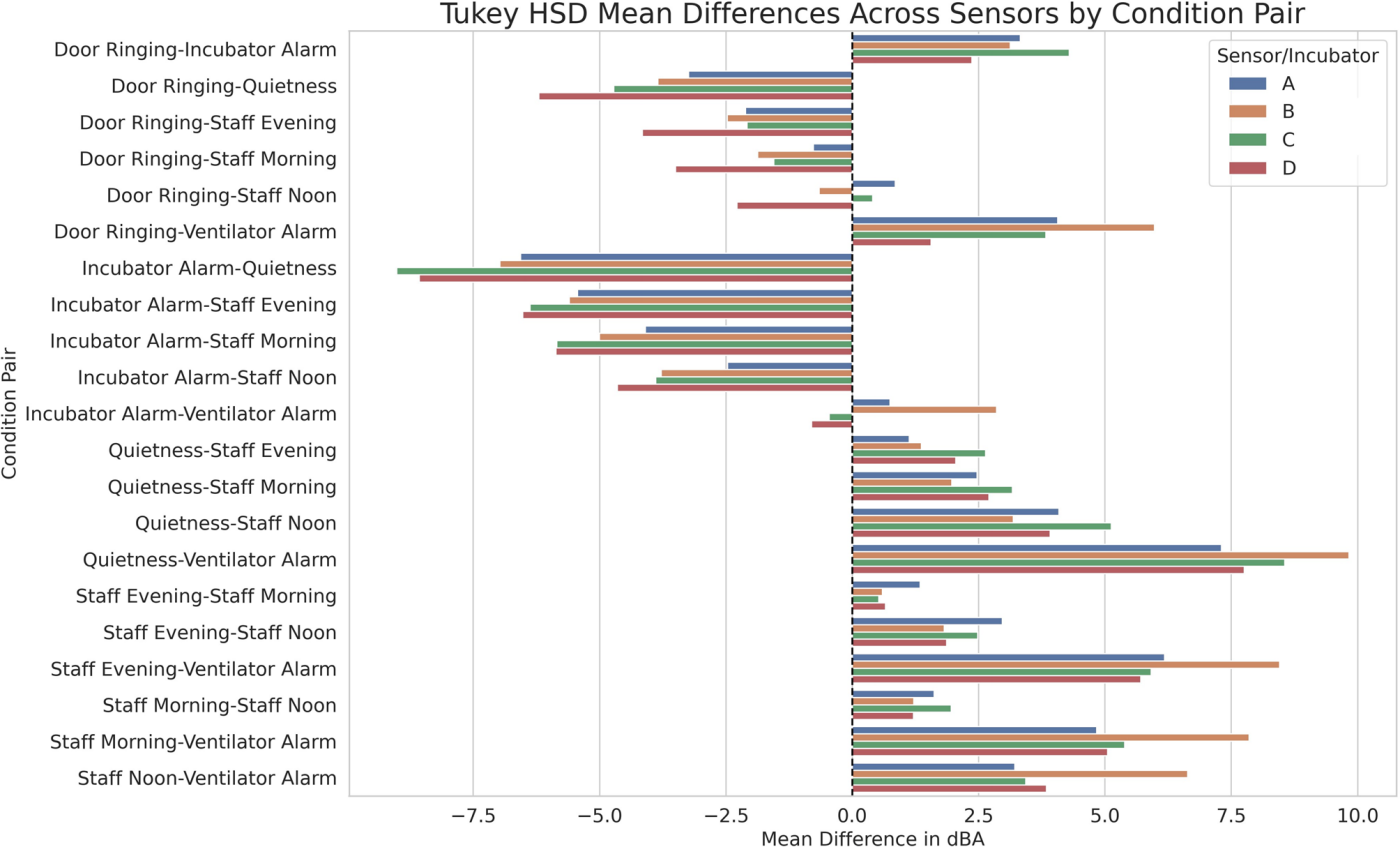
Tukey HSD results showing mean dBA differences across all condition pairs, separated by sensor (A–D). Each bar represents the magnitude and direction of acoustic contrast between two conditions.

To assess the consistency of acoustic differences across clinical scenarios and incubator zones, a multi-sensor Tukey’s Honest Significant Difference (HSD) analysis was performed. Figure 3 presents the mean dBA differences between all condition pairs, calculated independently for each of the four sensors (A–D). The visualization highlights both the directionality and magnitude of noise level differences attributable to specific clinical events. Across nearly all condition comparisons, the sensors demonstrated highly concordant patterns.

The contrast between ‘Quietness’ and ‘Staff Noon’ revealed a moderate increase in acoustic levels, with mean differences ranging from approximately + 2.5 to + 5.5 dBA across sensors, while comparisons with ‘Ventilator Alarm’ exhibited larger increases of + 5 to + 7 dBA. Comparisons involving ‘Door Ringing’ and ‘Ventilator Alarm’ demonstrated modest mean differences across incubator zones, typically ranging between 1 and 5 dBA, indicating that while both are high-noise events, their acoustic signatures can differ meaningfully across the NICU environment. Similarly, comparisons between ‘Incubator Alarm’ and ‘Quietness’ yielded a consistent mean difference of approximately − 6 dBA across all sensors, confirming the pronounced acoustic impact of alarm events in raising sound levels within the NICU environment relative to quietness periods. The overall consistency in pairwise contrasts across the four spatially distributed sensors reinforces the robustness and generalizability of the observed acoustic trends.

The classification performance of three supervised machine learning models—RF, XGBoost, and SVM—in classifying NICU conditions based on multi-sensor acoustic data is summarized in Table 2.

**Fig. 4:**
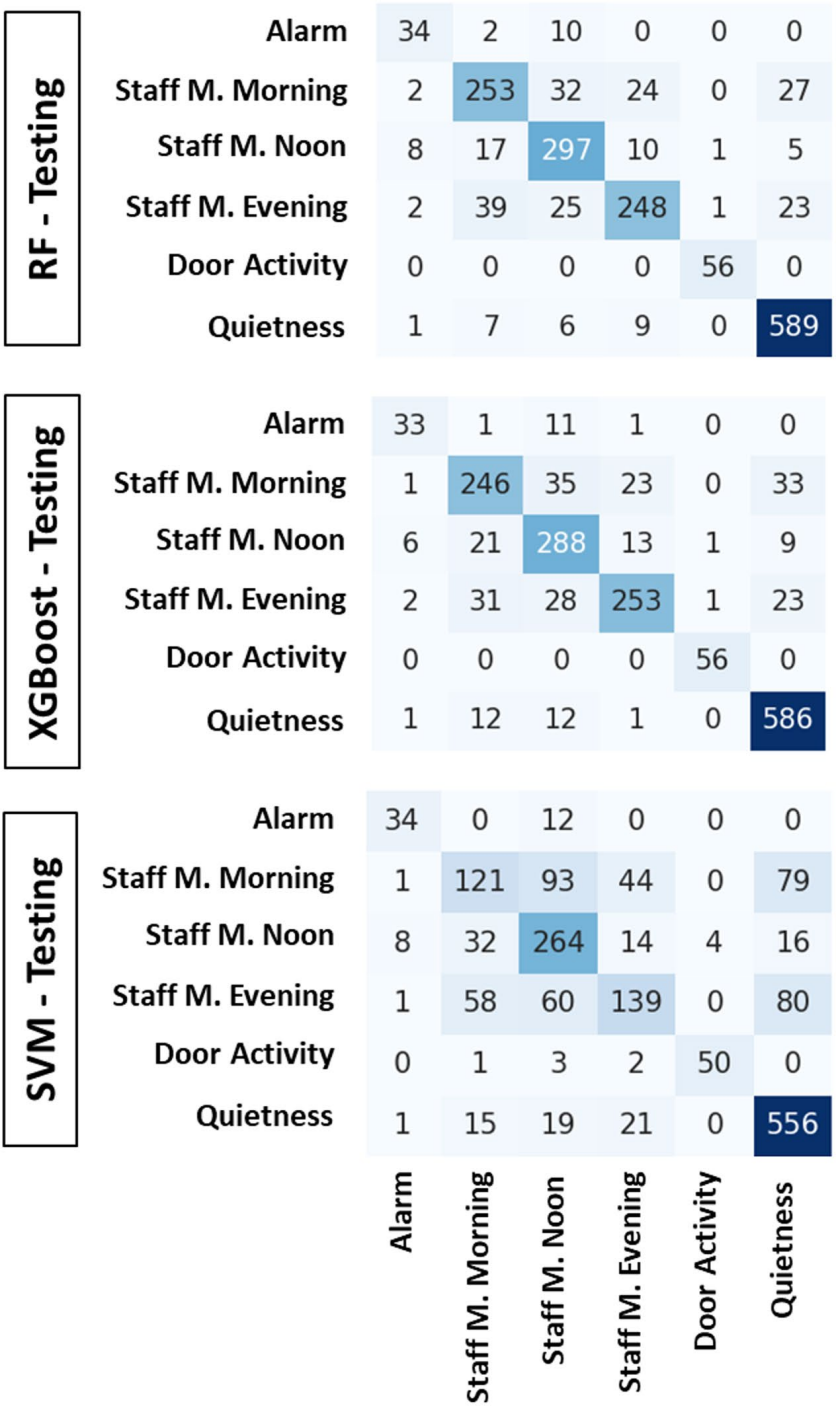
Confusion matrices for NICU acoustic event classification. RF, XGBoost, and SVM classifiers evaluated on the testing dataset are shown from top to bottom.

Among the evaluated models, RF yielded the highest overall accuracy at 85.47%, closely followed by XGBoost at 84.61%. The SVM classifier exhibited a substantially lower accuracy of 67.36%, reflecting challenges in generalizing across multiple event categories. Figure 4 presents the confusion matrices for the Random Forest, XGBoost, and SVM classifiers evaluated on the NICU acoustic event testing dataset. All models demonstrate strong performance in correctly identifying “Quietness” and “Door Activity” events, consistent with the high F1-scores reported. Misclassifications are primarily observed among “Staff Movement” classes across morning, noon, and evening periods, reflecting the acoustic similarity of these conditions within the NICU environment. The SVM model shows increased confusion among staff movement categories compared to ensemble models, which maintain clearer separation between these classes.

All models demonstrated excellent performance in detecting door activity and quietness. For these two classes, RF and XGBoost both achieved F1-scores above 90%, driven by high precision and perfect recall in the case of door events. Quietness was also reliably classified, with RF achieving an F1-score of 93.79% and XGBoost closely trailing at 92.79%. These results highlight the distinct and consistent acoustic signatures of both passive and transitional states in the NICU. Model performance varied across staff movement conditions. While noon-related activity was classified with the highest fidelity—reaching an F1-score of 83.90% for RF—morning and evening staff movement proved more challenging, particularly for the SVM model. SVM yielded markedly reduced F1-scores of 42.83% and 49.82% for morning and evening periods, respectively, primarily due to lower recall rates (35.80% and 41.12%). In contrast, ensemble-based classifiers maintained better robustness, with RF achieving up to 78.86% F1-score in the evening period. Across all models, performance on the Alarm category remained consistent, with F1-scores clustering between 73% and 75%, reflecting a reliable ability to identify equipment-generated acoustic events despite minor inter-model variation.

## Discussion

This study provides a comprehensive, high-resolution analysis of the acoustic environment in a functioning NICU, integrating spatially distributed sensors, statistical modeling, and machine learning to assess noise patterns with both temporal and spatial granularity. While our findings validate earlier reports of elevated NICU noise levels^4,6,10–16^, they extend prior work by explicitly linking sound intensity to specific clinical events, architectural zones, and operational time blocks—dimensions rarely explored concurrently.

**Table 2:** Classification metrics (%) for decoding NICU conditions from sensor data across three machine learning models. The reported values include precision (Prec), recall (Rec), and F1-score (F1) per class, with overall accuracy.

Event	Metric	RF	XGBoost	SVM
Alarm	Prec	72.34	76.74	75.56
	Rec	73.91	71.74	73.91
	F1	73.12	74.16	74.73
Staff movement morning	Prec	79.56	79.10	53.30
	Rec	74.85	72.78	35.80
	F1	77.13	75.81	42.83
Staff movement noon	Prec	80.27	77.01	58.54
	Rec	87.87	85.21	78.11
	F1	83.90	80.90	66.92
Staff movement evening	Prec	85.22	86.94	63.18
	Rec	73.37	74.85	41.12
	F1	78.86	80.45	49.82
Door activity	Prec	96.55	96.55	92.59
	Rec	100.00	100.00	89.29
	F1	98.25	98.25	90.91
Quietness	Prec	91.46	90.02	76.06
	Rec	96.24	95.75	90.85
	F1	93.79	92.79	82.80
Overall accuracy	—	85.47	84.61	67.36

The spatial heatmap in Fig. 1, generated through interpolated multi-sensor readings aligned with the architectural layout, revealed persistent noise concentration around incubators A and B, particularly near pulse oximeters and medical gas outlets. These findings align with observations by Fortes-Garrido et al.^17^ and Zamberlan-Amorim et al.^18^, who noted elevated noise near supply zones and room entrances. However, our study advances the field by providing a three-dimensional acoustic map, offering clinicians and architects an intuitive visual reference to guide spatial reorganization in noise-prone areas.

The statistical analyses presented in Fig. 2A and B demonstrate that all four sensors consistently detected significant dBA elevations during staff movement and alarm events, relative to quietness periods. Notably, mean sound levels during ventilator alarms exceeded 58 dBA—well above the American Academy of Pediatrics’ recommended ceiling of 40 dBA^6^. These values are consistent with the ranges reported by Bertsch et al.^8^ and Lasky and Williams^14^. Moreover, by stratifying staff activity by shift, our results reveal that the noon period bears the highest acoustic burden (*p* < 0.001)—a temporal detail underreported in previous research^9,23^. The observation that incubator door openings consistently produced high sound levels may carry implications for equipment design. Specifically, these findings suggest that manufacturers could explore quieter latch mechanisms or noise-dampening materials to reduce unnecessary acoustic disturbances in close proximity to the infant.

Our machine learning analysis (Table 2) adds a practical layer to the acoustic noise mapping in NICUs by demonstrating the feasibility of automated event classification from environmental acoustic data. The Random Forest classifier achieved 85.5% accuracy, with high F1-scores for quietness (93.8%) and door activity (98.2%). These results provide a prototype classification benchmark, extending beyond conceptual AI applications described by Hernández-Molina et al.^22^. Although the current study was performed offline, the high classification performance combined with the low computational load of ensemble models aligns with the trajectory of sensor-based predictive analytics in neonatal care^10,11^, offering a foundation for future near-real-time NICU monitoring system development. For real-time deployment, the system could operate using a rolling window approach (e.g., 30–60 s windows with partial overlap) to detect events within approximately 1 min of onset while balancing feature stability and latency. Given the low inference time of Random Forest and XGBoost, continuous updating of classifications is feasible without introducing substantial processing delays on edge devices or hospital servers.

The ~ 1-minute detection delay and 85.5% accuracy indicate that the proposed system is not designed to replace existing clinical alarm systems or respond to emergency conditions. Instead, the classification model may support longitudinal acoustic surveillance, environmental quality monitoring, or flagging of recurring non-urgent disturbances (e.g., excessive door activity or staff movement patterns). These insights can guide architectural redesign, noise source mitigation (e.g., latch dampening), or scheduling interventions, rather than triggering immediate medical actions.

Classification of acoustic events in real time can support several practical clinical use-cases in the NICU^20–24^. For example, frequent door ringing or staff movement during quietness periods may indicate excessive disturbances, prompting workflow adjustments to reduce noise exposure during critical infant rest times. Detection of ventilator alarms can enable automated logging and escalation if alarms persist, supporting alarm fatigue mitigation strategies. Identifying prolonged quietness can confirm effective noise control interventions. In addition, real-time acoustic classification enables automated heatmaps of high-risk acoustic zones and timely alerts when thresholds are exceeded, facilitating rapid staff awareness and targeted interventions without manual monitoring. These benefits align aligns with recent calls for multidisciplinary approaches to noise control, including architectural redesign^21^, staff behavior training^7^, and intelligent environmental management systems^10,22^. Our results lay the groundwork for future integration of real-time acoustic classification with practical mitigation strategies, such as threshold-based alerts for excessive noise, clustering of non-urgent care tasks to reduce repeated disruptions, and adaptive workflows aimed at minimizing hazardous sound exposure around vulnerable infants.

We acknowledge that the high-density, attached-bathroom configuration of our NICU may affect generalizability. However, the classification models are not architecture-dependent and can adapt to different NICU layouts (e.g., single-family rooms or open bays) by retraining on site-specific acoustic data with labeled events. Similarly, spatial mapping can be adjusted by repositioning sensors to reflect the geometry and functional zones of each unit, allowing identification of local high-risk noise sources. Because the models rely on features derived from relative acoustic patterns rather than absolute spatial layouts, the approach can flexibly extend to diverse NICU architectures while providing actionable noise profiling tailored to each clinical environment.

Manual annotation combined with direct mitigation planning is well-suited for small-scale or one-time assessments, particularly where staff can use domain knowledge to identify and address key noise sources. In contrast, the approach presented in this study is more appropriate for settings where ongoing environmental monitoring is needed—such as for generating noise exposure heatmaps, tracking long-term acoustic patterns, or automatically flagging recurring disruptive events without constant human oversight.

Dynamic closed-loop systems using reinforcement learning could eventually optimize workflows, alarm thresholds, or staff movement to reduce acoustic stress without affecting care. Coupling acoustic maps with wearable infant stress sensors may enable real-time linkage between noise and physiological responses for more responsive, infant-centered control.

## Conclusion

This study presents a robust framework for real-time acoustic monitoring in NICU environments, integrating spatial noise mapping, statistical inference, and machine learning-based event classification. By consistently identifying high-risk acoustic zones and associating specific noise patterns with clinical activities and time blocks, the results underscore the importance of spatial design considerations and workflow optimization in neonatal care units.

The high classification performance of ensemble models—exceeding 85% accuracy in classifying clinical events based solely on acoustic data—demonstrates the feasibility of deploying low-cost, sensor-driven systems for real-time environmental awareness. These findings support the future integration of intelligent alert mechanisms and adaptive management protocols to proactively mitigate noise exposure.

## Data Availability

The datasets analyzed during the current study are available from the corresponding author upon reasonable request.
